# “*You see, we women, we can't talk, we can't have an opinion*…”. The coloniality of gender and childbirth practices in Indigenous Wixárika families

**DOI:** 10.1016/j.socscimed.2020.112912

**Published:** 2020-05

**Authors:** Jennie B. Gamlin

**Affiliations:** UCL Institute for Global Health, 30 Guilford St., London, WC1N 1EH, UK

**Keywords:** Indigenous, Maternal health, Colonialism, Childbirth, Pregnancy, Health inequality, Gender

## Abstract

How women make decisions about care-seeking during pregnancy and childbirth, is a key determinant of maternal and child health (MCH) outcomes. Indigenous communities continue to display the highest levels of maternal and infant mortality in Mexico, a fact often accounted for by reference to inadequate access to quality services. A growing body of research has identified gender inequality as a major determinant of MCH, although this has rarely been situated historically in the context of major social and epistemological shifts, that occurred under colonialism.

I used a feminist ethnography to understand the structural determinants of Indigenous maternal health. I drew on research about the colonial and post-colonial origins of ethnic and gender inequality in Mexico and specifically the Wixárika Indigenous region, in order to identify the different ways in which women have historically been disadvantaged, and the processes, situations and interaction dynamics that emerged from this. Sixty-four Wixárika women were interviewed while pregnant, and followed up after the birth of their child between January 2015 and April 2017. These data were triangulated with structured observations and key informant interviews with healthcare providers, teachers, community representatives and family members.

The findings suggest that gender inequalities were introduced with the colonial system for governing Indigenous regions, and became naturalised as Wixárika communities were increasingly integrated into the Mexican nation. The associated structures of marriage, community and interpersonal relationships now operate as forms of institutionalised gender oppression, to increase Indigenous women's vulnerability, and influence decisions made about care and childbirth. Ethnographic data analysed in historical context evidence the continuity of colonial forms of inequality, and their impact on wellbeing. While welfare and health programmes increasingly aim to address gender inequality on social and relational levels, by rebalancing gendered household dynamics or empowering women, the historical and colonial roots of these inequalities remain unchallenged.

## Highlights

• Evidences link between colonialism and gender inequality in Wixárika communities.
• Reveals how maternal outcomes are influenced by gender violence and polygyny.
• Reproblematises maternal and infant death as the Afterlife of Colonialism.
• Argues that decolonial approaches can improve indigenous health.

## 1 Indigeneity, coloniality and maternal health

Globally, Indigenous people's health is ‘invariably lower than that of the overall population’ (WHO, 2013). In Latin America, the colonial political and social structures that went on to form the basis of independent nation states have not been favourable to equality between ethnicities (Fisher and O'Hara, 2009; Valeggia, 2016). In the case of Mexico, deep inequalities established during the colonial era were strengthened throughout the process of nation-building. This entailed the promotion of a national identity based on the mestizo (mixed-blood) population (see for example Arizpe, 2015; Kellogg, 2005), creating ‘racial’ hierarchies from ethnic difference. As in other parts of Latin America, national aspirations to ‘whiteness’ represented by mestizo and European lifestyles have become determining factors in the delivery of institutional healthcare (Guerra-Reyes, 2013), to the exclusion of Indigenous-specific concerns and practices. During recent decades that have seen advances in Indigenous rights, these historical structures have continued to undermine the wellbeing of Indigenous people (Stephens et al., 2005). Although constitutional changes have led to some advances in political autonomy for Indigenous peoples in Latin America, racial equality and social wellbeing have not followed; these remain dependent on policy and institutions at a national level. It is the continued political, social and cultural dominance of Indigenous populations by nation states that has led this relationship to be theorised as *coloniality*.

Throughout Latin America, Indigenous communities often evince deeply embedded gender inequalities, which combine with ethnic vulnerabilities to adversely affect health outcomes along this intersect (Valeggia, 2016). In Mexico, high Maternal Mortality Rates ‘plague the rural and Indigenous poor’ (Dixon et al., 2018). The states of Chiapas (MMR 68.1/100,00 live births), Guerrero (MMR 58.7) and Oaxaca (MMR 46.7), with their large Indigenous populations, score the highest on this indicator, in contrast to a national MMR of 38 (CIA, 2015). Infant and maternal mortality track the same fault lines, and municipalities with high infant death rates are predominantly Indigenous. As recently as 2005, the municipality of Mezquitic, where this study took place, had the second highest Infant Mortality Rate in Mexico: 76 compared to a national rate of IMR 13 in the same year (INEGI, 2005). Evidence also suggests that these rates mask considerable under-reporting (Gamlin and Holmes, 2018).

Mexican Indigenous communities have been poorly provided for in terms of health facilities. They are often less accessible than non-Indigenous regions, with weak transport and communication links. Generalised conditions of poverty and poor-quality education also mean that many Indigenous women are ill equipped to deal with obstetric emergencies, in terms of both accessing quality services and knowing when to seek care (Freyermuth and Argüello, 2010, Sesia et al., 2007). These are both key drivers of maternal and infant mortality on the supply side (Koblinsky et al., 2016). At a global level, we know that poverty and education are important determinants of institutional delivery (Freidoony et al., 2018; Yaya et al., 2017), with higher rates of skilled birth attendance leading to lower mortality rates (Aminu, 2014). These are some of the findings that have informed global efforts to reduce maternal mortality. In Mexico, training traditional midwives, good surveillance in the form of the Observatory for Maternal Mortality (OMM), and political pressure to ensure health budgets reach the people they are intended for, are all considered good practice for the reduction of maternal mortality (Freyermuth, 2015). While there is general agreement among global health experts about the drivers of good maternal outcomes, there has been little or no consideration of the specificity of Indigenous populations, or the coloniality of care, implying that health service provision continues to operate though the imposition of a set of cultural and power structures and assumptions that are colonial in form. This means that global health governance and national health systems determine what is best practice for Indigenous populations, or exert biopower over them, in accordance with global norms. These include law, epidemiology and humanitarianism, which represent the continuity of colonial structures of power, implying a loss of health sovereignty and ‘control over the conditions and methods of care’, for Indigenous people (Morgensen, 2014). Although the ‘coloniality of global health governance’, as a concept, was largely developed in the context of North American settler colonialism, the structures and delivery of healthcare in Latin America are equally exclusive of Indigenous participation and knowledge systems. Policies are largely defined centrally, and often in accordance with proposals, such as Universal Health Care (UHC) and the promotion of institutional delivery, that are defined by global health governing organisations. As Smith-Oka notes, the top down approach of UHC ‘needs to be accompanied by an effort to *understand and overcome persistent economic and social hierarchies*’ (Smith-Oka, 2015: 15, emphasis added). Non-biomedical and non-Western childbirth practices are diverse, but birthing women are usually physically supported by another individual, as was the case elsewhere before the rise of biomedicine and obstetricians (Jordan, 1993). Wixárika women who give birth at home do so alone, or physically supported by a family member or spouse. However, institutional (local clinic or hospital) deliveries, and in particular those where the supine position is encouraged, eradicate the role of non-medical support and few hospitals allow entry to a partner during labour (Gamlin and Osrin, 2018). Ethnographic work with Mexican Rarámuri and Mayan communities has suggested that active male participation in childbirth can lead to greater gender equality and positive birth outcomes (Miller, 2009; Ortega Canto, 2010). Institutional exclusion of men from this role is just one example of how the coloniality of global health governance is impacting on Indigenous communities.

Critical ethnographies of maternal and infant death in Mexican Indigenous communities have consistently pointed to ethnic, gender and economic inequalities as risk factors for maternal and infant death (Freyermuth, 2004, 2015), and conceptualised these avoidable deaths as structural violence (Gamlin and Osrin, 2018; Freyermuth and Argüello, 2010; Smith-Oka, 2015). A decolonising approach adds a new perspective to this discussion by suggesting different problematisations from which to address maternal and infant mortality. To do this I extend Hartman's concept of the afterlife of slavery, which she describes as ‘skewed life chances, limited access to health and education, premature death, incarceration and impoverishment’ (Hartman, 2007: 6), into the context of coloniality. Davis (2019: 89) operationalises this concept to describe the fallout of pregnancy and prematurity that some Black women experience as: ‘suboptimal care that […] may result in part from the legacy of racist treatment during the antebellum period and in the afterlife of slavery’. I argue that Indigenous experiences of pregnancy and birth are the afterlife of colonialism, a legacy and mingling of the colonality of gender and healthcare.

## 2 Coloniality of gender

The ‘modern/colonial gender system’ sits at the intersection of race, class and colonialism (Lugones, 2008). By extending the idea that colonialism is an ongoing process, as opposed to a defined historical period (Quijano, 2000), Lugones describes the intertwining of race and gender that produced this system. Following the European invasion of the Americas, the initial centuries of this process happened in parallel to European state-making, leading to profound social and political transformations that Silverblatt (2009: ix) describes as ‘a revolution in the possible ways of being human’. In this paper I *re-problematise* Indigenous maternal and infant mortality from the perspective of the coloniality of gender, positioning this as a structure that disrupts processes of birth and reproduction, and limits the ability of families to prevent avoidable maternal and infant deaths. The resulting excess morbidity and mortality have become an afterlife of colonialism, as structures established under colonialism live on in the bodies of women and babies.

Gender is an almost universal structure that is formed relationally, through interactions between people, and through the actions and expectations of individuals and groups. These are supported by institutions and structures, such as the family, education system and the labour market (Connell, 2005; West and Zimmerman, 1987). Defined gender roles and institutions then become naturalised, and are embodied in the behaviour of men and women. In this manner the coloniality of gender has become invisible. Largely defined through cultural processes, including religious and economic organisation, Western colonial patriarchy reproduced itself within institutions such as marriage, kinship and the sexual division of labour. These institutions were then exported to colonial states, where Western structures reconfigured gendered identities in relation to those of the metropole (Mies, 1986; Wiesner-Hanks, 2008). Race was produced as part of the same process, with the ethnicity of Indigenous people *biologised* and positioned hierarchically in relation to that of Europeans. Lugones (2008: 2) describes the invention of ‘race’ as a ‘pivotal turn that replaces the relations of superiority and interiority established through [colonial] domination’, becoming an axis of oppression that is part of the system of coloniality.

## 3 Gender in history

Historical analyses of gender in Latin America largely agree that the colonial period and subsequent centuries saw the transformation of more egalitarian ‘parallel’ or ‘complementary’ gender systems (Fisher and O'Hara, 2009; Kellogg, 2005; Segato, 2015) into a binary and hierarchical structure. Unequal gender relationships resulting in violence are highly prevalent in Mexico (González-López, 2015; González Montes, 2009), particularly in Indigenous communities. Some research has questioned why gender violence is so prevalent, and begun to explore how historical processes have influenced gender identities in Mexico. The work of González-López (2015) examines ‘ecclesiastical justice’ and the legal apparatus in colonial and independent Mexico that were reflected in laws regarding incest. She describes how ‘women did not have rights as fully sentient autonomous human beings’ and notes that ‘nineteenth century legal commentators warned that husbands and wives could not be equal’ (p17). These legal structures were strengthened by Catholic values and later consolidated as ‘codes of honour and shame’ that defined rigid gender roles for men and women. Hierarchical gender relationships then became embedded in male honour, which itself served as a cause of violence against wives (Kellogg, 2005: 79). Mexican colonial and post-colonial history also reveals the intersection of gender and poverty. Kanter (1995: 609) describes how the Spanish authorities upheld traditional land tenure rights in central Mexico, which entitled women and widows to inherit land, but following independence, changes to land ownership became patrilocal, giving priority to men ‘of legitimate birth, native to the village’. By the mid-nineteenth century ‘examples of female land tenure hardly existed’ (Kanter, 1995: 613). This redefining of race and gender was central to post-revolutionary nation-building, and became reflected in cultural and social institutions – such as health, education and the family – that shaped modern Mexico.

Yet while gender is a historically embedded social structure, human memory is short. As Lugones (2010: 745) suggests, the ‘colonisation of memory and thus of people's senses of self … [their connection] to the spiritual world, to land, to the very fabric of their conception of reality, identity’ all formed part of the ‘civilizing transformation’ that occurred during colonisation. Early ethnographic work with Wixárika people suggests that at the time of colonisation their gender structure was considerably more equal. Unlike central and southern Indigenous communities, Wixárika people largely resisted colonial power until 1722. When Franciscan missionaries and colonial governance systems were finally established in the region, Wixárika people had a well-developed religious and ceremonial system, with gods and goddesses of equivalent importance in key mythological narratives and performances (Preuss, 1996; Weigand, 2002). Like all Mesoamerican ethnic groups, Wixárika people's lives were at the absolute service of their gods, and divinity also shaped their marital ties. Writing in 1902, Lumholtz described how women's status in the family was high, and although at the time of his research political positions were occupied by men, ‘in ancient times, tradition says, women held these offices’ (1902: 246), suggesting that a patriarchal system took over at some point between 1722 and 1900. Early descriptions of marriage make no reference to formalised polygamy, but do suggest that men in positions of authority began to take on second and subsequent wives, either as ‘gifts’ from colonial superiors (Weigand, 2002: 58), in line with the privileges conferred upon Spanish and mestizo governors (Weigand, 1992: 124), or as domestic helpers known as *tenanchas*, whose role subsequently merged into that of mistress (Lumholtz, 1902: 246). That marriages were often non-monogamous and never strong is mentioned by several studies (Mata Torres, 1982; Weigand, 2002), yet Lumholtz further elaborates, suggesting that ‘while *guarded by religious beliefs* they were far more secure than now, when nothing but the fear of corporal punishment, lashes and the stocks in prison, restrains the people from indulging their fancy too freely’ (1902: 96, emphasis added). Today, Catholicism only peppers Wixárika religious beliefs and practices, although it has left a considerably stronger impression on social and moral order, transforming gender dynamics through the introduction of systems of corporal punishment and male superiority.

The relevance of this discussion to maternal and infant mortality lies in the urgency of newly problematising longstanding and intractable problems. Current programmes for addressing maternal and infant mortality in Indigenous contexts such as the Madrinas Obstétricas programme -which supports pregnant women so that they were able and more likely to access clinical care during pregnancy and for the birth of their children-have had a limited impact, and do not address Indigenous wellbeing from a gender or race perspective, but largely focus on increasing the proportion of institutional births (Freyermuth, 2015). This has additionally contributed to the ‘routinisation’ of care within an overburdened system (Smith-Oka, 2013). Within global health, gender has overwhelmingly been addressed within neoliberal (Wilson, 2015) and instrumental (Gideon and Porter, 2016) development frameworks that largely reinforce structures of gender inequality. Examining gender as part of a process of colonialism will provide pointers for critically redefining these inequalities, evidencing potential interventions and processes aimed at addressing structural gender inequality and its impact on health and wellbeing.

## 4 Methods

### 4.1 A critically positioned feminist ethnographic methodology

Critical research engages with problematic issues that might otherwise ‘slip into the convenient silences of social and economic policy’ (Hooper, 2013: 201). To challenge this, Hooper (2013) argues for ethnographies of health that are ‘in-depth, long term, fiercely argued and unafraid of offending’ (204). While gender inequality has been clearly identified as a structural determinant of maternal health, its institutionalisation at a community level and articulation around coloniality is rarely expressed in relation to health.

This study aimed to understand the coloniality of gender in Wixárika communities, and how this impacts on maternal and infant health. I used the methodology of feminist ethnography to ‘hone … in on people's statuses, the different ways in which (multiple) forms of privilege allow them to wield power or benefit from it, and the forces and processes that emerge’ (Davis and Craven, 2016: 9). Feminist ethnography does this by analysing personal narratives, and specifically seeks to identify how gendered power dynamics operate to impact on people's experiences. In this case I sought to understand how gendered power influences birth outcomes. This is also a critical ethnography, seeking to denaturalise assumptions about social structures to ‘displace the lines of the obvious’ (Fassin, 2013: 123). To practise this, feminist ethnography involves building up understanding by layering data gathered using different methods, and here I evidence the permanence of the past in the present, by combining ethnography with ethnohistory and using an interpretative framework for analysis that draws on the theory of coloniality. In this paper I present data that illustrates how gender relations have become institutionalised in Wixárika communities, and I analyse historical circumstances from a coloniality of gender perspective to visibilise the impact that colonial and post-colonial governance and institutions had on previously more egalitarian gender relationships.

### 4.2 Data collection methods and context

This study used interviews and ethnographic methods to understand the structural determinants of maternal and infant health in the Indigenous Wixárika community of Xirawe (not its real name), Jalisco state, northwestern Mexico. The governorship comprises *pueblos* (small highland towns) and *rancherías* (extended family farms), spread across a valley. The two main *pueblos* are accessible via unpaved road, and lie two to four hours from the municipal town and hospital. Each highland town has a Ministry of Health clinic with a fully qualified doctor (MD), a travelling doctor (TD) and a student doctor (SD), under Mexico's *Seguro Popular* health system for the uninsured. Officially, the MD attends the clinic with a nurse from the 11th to the 30th of each month, while the SD is present from the 20th of each month for 20 days, running the clinic alone from 1st to 10th of each month. This system of healthcare provision is repeated throughout the Indigenous region. In contrast, lowland and mestizo towns have a regular and constant service. The Wixaritari (plural form of Wixárika) also have a traditional medicine system, which locates illness in the social and supernatural realm. There is no tradition of midwifery, but around 60 per cent of births occur at home, with women giving birth alone, or accompanied by a family member (Gamlin and Osrin, 2018). Politically, the autonomous governorship is a dependency of Mezquitic Municipality, from where services such as health, education and the *Prospera* welfare programme are coordinated. *Prospera* is a government-run cash transfer scheme, which gives women bi-monthly cash payments for each child enrolled in school. Higher amounts are paid for female children. To be eligible women are also required to attend health promotion and exercise sessions at the local clinic. Failure to comply with these conditions leads to payments being cut and the MD at the local clinic is responsible for enforcing the conditionality. The community has political autonomy for internal issues that are not the concern of the state. Therefore local authorities preside over cases of marital infidelities, violence or abuse and conflicts over land ownership, community residence or theft, but they have no voice in issues related to health, education or the Prospera programme.

### 4.3 Sample recruitment and data collection

I have worked in Xirawe since 2009, and for this study recruited eight bilingual research assistants. We used semi-structured interviews to gather data during pregnancies and narrative interviews after the birth of their child, asking about birth experience with prompts about treatment by health providers, interventions or complications and expanding to discuss issues such as marital relationships. In addition, I gathered observational data and conducted informal interviews with health providers (8), teachers (3) and key community and family members (approximately 15). These were gathered in a field diary or audio recorded.

At a first stage, women were recruited through the local General Assembly meeting, where we also obtained support and approval for the project. Following recruitment, the eight research assistants attended community meetings, to request the participation of pregnant women. We sought to interview all women who were pregnant during the twelve-month period of January–December 2015. Each of the eight research assistants travelled on foot to individual *pueblos* and *rancherías* and women were largely identified by word of mouth. A convenience sample of sixty-four pregnant women aged 13–39 (mean age 24.5 years) were interviewed at baseline by one of the eight research assistants, using a semi-structured interview schedule. These data were gathered by hand in Spanish and generated socio-demographic data and details of birthing intentions. All women who were known to be pregnant and could be found at home at the time of the study were invited in person, to participate. Based on data from Mexico's National Institute of Geography and Statistics, I estimate approximately 35% of pregnant women in the governorship joined the study (INEGI, 2015). The women who did not participate may have been away from home when the interviewers called, chose not to be interviewed (8 refusals were documented), or did not disclose their pregnancy to anyone.

In-depth follow-up interviews were conducted after the birth of a child. I conducted each of these interviews with the assistance of one of the eight bilingual research assistants. A set script in Wixárika was used to explain the purpose of the study and request consent to record and later publish data anonymously. All Wixárika names, including place names, have been changed. Consent was given verbally, and ethical approval was provided by University College London (UCL) Ethics Committee. The sample is likely to be weighted towards women who live in the highland towns, and therefore potentially biased towards those who have greater access to education, transport and health services.

### 4.4 Analytical strategy

In order to achieve an accurate representation of voices, interviews were audio recorded, transcribed directly into Spanish and double-checked for accuracy by a bilingual research assistant. These data were discussed in depth with the team of eight research assistants, who clarified and provided context for the interview data. Post-interview discussions were recorded in a field diary, together with observations, and these were transcribed directly into Word documents. I conducted an inductive thematic analysis of all data using N-Vivo version 8, to manually generate codes and sub-themes. Following the general practice of grounded theory (Strauss and Corbin, 1990), data that were coded together included interview transcripts and observational field notes. These were then collapsed into twelve overarching general themes so that they could be analysed individually in more detail. This enabled various simultaneous views of specific data, to illuminate the complex and multiple meanings of statements where indirect references are made to issues such as unequal partnerships. N-Vivo also enabled analysis of data by group of informants, allowing stratification by informant type, triangulation between key informant type, post pregnancy interviews and observational data and to isolate specific interviews. In one case this provided multiple descriptions of a complex birth, in other cases I was able to compare health care provider's views with those of community members around controversial issues. All data described above contributed to the thematic analysis and subsequent theoretical interpretation. In this paper I discuss data that relate to gender equality in intimate partnerships and within the community.

## 5 Findings

### 5.1 Gender inequality as a social structure

#### 5.1.1 Marriage and vulnerability

Most of the women interviewed were married, some for the second time, some as second wives and some to a man who had later taken a second wife. It is rare to find a couple who have maintained a single partnership throughout their lives, this implies that women usually tolerate a string of infidelities, being one of several wives or being abandoned by the father of their children. Few Wixárika marriages would be recognised by Mexican law; instead they are witnessed by local authorities or exist as common-law partnerships, which under Wixárika law can be polygynous. Marriage often occurs soon after menarche and, if the couple are still minors, subject to the approval of one or both sets of parents. It was common to hear the phrase *nos juntaron*, literally meaning ‘they joined us’. In some cases this happens because young couples are spotted displaying physical affection or thought to have consummated their relationship, and – although this is less common now – some young girls and boys are ‘joined’ into arranged marriages.

Polygyny, adolescent marriage and forced marriage can each make a woman vulnerable, and often they occur together. I asked Ester why a woman would marry a man who already had a wife. She responded by telling me her own story, of being forced to become the second wife of Obaldo, an older man:

‘When I arrived at the Agency [local government office] my whole family was there, as well as the authorities, Obaldo and his wife. They told me that I had gone with Obaldo to the field after the dance, and that because of this we had to end the relationship or they would make us marry. Because I was a minor I didn't have the right to say anything [at the meeting].

I began to cry and said I had done nothing, and wanted nothing to do with Obaldo. Then my uncle spoke and said that since I had no parents, someone needed to be responsible for me, and if I had done what these people were saying, then I should marry Obaldo. Anyway, no-one in the family could support me financially, and since this man was offering to do it, then it would be best if I went with him.’

Parental complicity in forced or early marriage can be a means of lightning the household burden or avoiding shame. Whichever is the case, it often leads to pregnancy at a young age and frequently also to intimate partner violence, as Jessica described:

‘She [Mum] supported me and said that the baby was on its way and whatever the case this wasn't her [the baby's] fault, and she had to be born. And well, that was what most helped me and gave me strength. But I did have a bad time, my husband and I argued a lot and he said “was it really his [baby]? … Maybe it was another person's”, and then he would hit me.’

Early marriage and pregnancy led to Jessica abandoning school aged fourteen, after her husband told her that ‘study was not for women’. During their interviews many women recounted stories of beatings, citing jealousy or a neglect of domestic duties as the reasoning behind these.

None of the women in polygynous marriages described equitable or amicable arrangements. Some co-wives lived in different rooms around a shared patio. Others lived very separate lives, with one wife in a town house, and the other caring for the farm. Living arrangements had implications for childbirth. Estela lived alone in a rented house with seven children, who she maintained on the small *Prospera* welfare payment. When I asked about her husband, who lived with his other wife, she replied that ‘he only turns up to make me pregnant and take money from me’. She had been alone in her father's ranch when her baby was born. Fortunately her father arrived in time to help with the birth.

Alicia gave birth alone, while her husband slept in the room next door with the other wife. She was lucky: her neighbour heard her, and came in time to catch the baby and cut the cord. Maria was less fortunate. She had been alone in a distant ranch with four small children when her baby was born, and exclaimed that her husband only appeared on rare occasions, when he needed a place to sleep, leaving her penniless and pregnant.

#### 5.1.2 Violence

Structural gender inequality is manifest in unequal partnerships between men and women. Polygyny is one manifestation of this; intimate partner violence and sexual abuse are others. Abril, whose grandson lived for only a week, explained what had happened to her daughter, Jovita:

‘The man didn't come at any point in her pregnancy, then she gave birth. I didn't confront the man either, and Jovita said to me, “Since he abused me he hasn't spoken to me … I don't know why he hasn't come. I think that he just did this to humiliate me and I feel bad.” And well, then the baby was born and all this happened. He didn't even accept that the baby was his.’

Several narratives described the convergence of young marriage and physical violence as negatively affecting pregnancy and childbirth outcomes. Maribel became pregnant at thirteen and moved to her husband's family home. She gave birth at seven months' gestation, in the cabin of a truck, outside the clinic. When I asked her about her birth, she described going into labour late at night, and the clinic being closed. Her in-laws also spoke to me about the birth, emphasising how the clinic would not attend her, as she was not enrolled with the *Seguro Popular* health insurance programme. After listening to both accounts, Lola – my research assistant – described how on the night that Maribel's labour began, she had turned up at Lola's home, having been beaten by her husband for the umpteenth time. Lola tried to persuade her to leave her husband, and to stay with them until her baby was born, but before the end of the evening her husband had come for her. Maribel's labour pains began shortly afterwards, and Lola was convinced that her premature labour was a direct result of the ongoing beatings that Maribel had been receiving from her husband.

### 5.2 Political and religious structures

‘You see we women, we can't talk, we can't have an opinion’, I was told on the day that I presented this project to the General Assembly meeting. Wixárika marriage patterns are embedded in a patriarchal structure of authority that rarely sides with women. Often asked to adjudicate in cases of infidelity or interpersonal violence, traditional authorities will usually seek adherence to a system that privileges men. Alejandra, an MD in the highland clinic, explained to me that she sees two or three cases of intimate partner violence every month, but is virtually powerless to help. If the case is referred to traditional authorities, they may punish a husband by asking him to pay a fine, for example two cows, to his wife or her family, but more often than not, this is on the basis of the wife unconditionally returning to her duties in the family home. Maribel experienced a visible onslaught of violence, from before the birth of her child. In relation to this case a key informant told us, ‘There are none of us that have the guts to take on her husband and the authorities, to protect her.’ Alejandra explained to me that women who have been abused may seek legal support outside the community, although this usually results in the woman being unable to return, and losing her social support system.

### 5.3 Gender's intersection with poverty

Poverty impacts differentially upon men and women. Xauxeme left school after finishing primary education to work in the *campo*, as he didn't have a dad. ‘Here it's difficult for women to find where to get money,’ he tells me. Women who do not have a partner or family are always the most vulnerable to both poverty and abuse and, as was the case for Ester, becoming a second wife can be preferable to being on your own. Orphanhood leaves any child vulnerable, but girls experience the added risk of being abused by family members, married off to lighten the burden on their adoptive family, or having to provide marital duties in exchange for some form of support. Rosalía, who was twenty-two when we met, was sent at a very young age to live with a much older farmer, who used her for sex and domestic help. Two of her children died while she lived with him and before she was able to escape. She then married a man in a different town and soon fell pregnant again, only for her husband to leave her before the baby's birth. When we met she had entered into an arrangement with the grandfather of her new baby, whereby she cared for his valley home in exchange for somewhere to live, while he controlled her private and public life.

For many women, *Prospera* payments were their only income. These conditional cash transfer schemes are policed by the clinic MD. To receive them women must attend regular health check-ups and exercise sessions, while children must attend school, but it is often the case that the most vulnerable women, like Rosalía, are unable to comply with them. This was also the case of María, who gave birth while alone with her small children in a very damp house. ‘Migrating for work is not an option when you are alone with five children,’ she told me. As was often the case, the oldest child remained at home to help care for younger children, and without school enrolment she was not entitled to welfare payments. María's Spanish was very basic and for this reason she refused to attend the clinic, so neither she nor her children had ever received healthcare of any kind.

### 5.4 Men's role in childbirth

As women narrated their experiences of childbirth, many spoke of the important role their husband took in assisting the birth, taking them to the clinic and providing moral support. Although Wixaritari do not have a tradition of midwifery, a close family member may support then in diverse ways during labour and spiritual or other assistance is often provided by a *mara'akame* (shaman). Of the sixty-four women we followed up post-partum, twenty-eight gave birth at home with assistance, and five gave birth completely alone. Childbirth is not considered a woman-only zone, and a male *mara'akame*, father or husband is often present. Ofelia, who gave birth in her home in the valley community, described how ‘My husband held me around my waist, above my stomach so I could lean on him’ while her baby was being born; Nayeli described how she gave birth ‘crouched down holding onto a *soga* [rope tied from a beam in the ceiling] and my husband caught the baby and cut the umbilical cord’.

During labour and in emergency situations men are critical to the decision-making process, and this may be delayed in the absence of a husband. Mijares felt that he was not able to support his wife, Zenaida, through the birth of their child and insisted they go to the clinic. Highland clinics have come to recognise the important role that men play in childbirth, and allow men to accompany their wives during labour. In complete contrast, men are excluded from the birthing process in urban public hospitals. Mijares explained how this decision was made:

‘When I first spoke to her she didn't want to go [to the clinic], and so I said to her, “It's better if we go because maybe something could happen here, it's not safe, it's better there because there is an ambulance so that they can send you [to the hospital].” So that's why I took her to the clinic.

Women will often delay making a decision, financial or otherwise, either in the hope that their husband will make that decision first, or, if he is absent, that he will return in time to make the decision. Either scenario ultimately leads to a delay in seeking care. Luisa's husband was absent when she went into labour. They were in a valley community, and had been waiting for him to arrive before returning to the highland town, where the health clinic is located. But he didn't arrive so they remained in the valley. Sarahi, Luisa's mother, described the birth:

‘It's because the baby was born feet first, that's why it died … I pulled its little foot, which was already floppy. We were lucky Luisa didn't die too. I told her husband to look after her and he went [to work as a migrant labourer], telling us that he would be back on the 20th, but he didn't get back … and well, I didn't know what to do, the baby died because it was born feet first and got stuck. Later when her husband came I said to him, “Take her to the clinic so that they give her a drip because she has lost a lot of blood,” I said to her husband. But he didn't take any notice of me.’

As well as decisions about where to give birth or how to respond in an emergency, men also very often have the final say in financial decisions. Chavela, who was pregnant with her eighth child, returned to her valley community from the hospital, where she had been for a scan, with ythe news that her baby was in a transverse position. She told her husband, Epigmenio, that doctors had said she should stay near the hospital until her baby was born, and she asked him for permission to return to the town. Epigmenio replied that they had no money to pay for the fare and there was no-one to care for their children, so Chavela stayed. When her labour began they called for a shaman, who came and turned the baby by massaging her stomach. Although the baby was born well, Chavela's mother, Alicia, arrived shortly after the birth to find her daughter in a pool of blood. She told us:

‘He was sat here [Epigmenio], with his head like this [inclined forward]. Then I got up, and I said to Epigmenio, “And what's happened to her … how long has she been like this?” Then I touched her and moved her and she was cold, her hands were hard as were her legs. And I'm like, “How is it possible that he left her like that? Wasn't he looking at her? Why didn't he ask her what was wrong?” Because he said to me that when she fell she cried “Ay!” Why didn't he call for help immediately?’

It took Alicia some time to find a vehicle that was able to take them to the clinic, as the first driver she found was waiting for someone else, and half an hour passed before Chavela was carried to the truck. By the time they arrived at the clinic an hour away, Chavela had died.

## 6 Discussion

Gender inequality is embedded in Wixárika social arrangements of marriage, kinship and governance, making it a structure of inequality. This structure is reinforced through institutions within and outside Wixárika communities. Ethnohistorical data suggest that these arrangements can be traced back to colonial and post-colonial events, indicating the *coloniality* of contemporary Wixárika gender structures.

Wixárika women are disadvantaged through marriage, because they often have to share their husband with one or more women, either within a polygamous marriage or extramaritally. There is no evidence to suggest that formal polygamy was practised in pre-invasion Wixárika society, but early ethnography (Lumholtz, 1902) and ethnohistories (Weigand, 2002) suggest that it was introduced alongside European political and religious structures. Its normalisation and acceptance within contemporary society could reflect the increasing superiority of men. All early ethnographies document how Wixárika lives were at the absolute service of gods, and all aspects of life revolved around ensuring the fertility of land and people. Although certain rituals still require monogamy, divine punishment is not feared, as it was among the generations that Lumholtz knew and polygamy can in fact be supported by authorities. More widely within Mexico, it has been suggested that the proliferation of polygamy was linked both to post-invasion ethnocide, which resulted in a shortage of men (Rovira, 1997; Pescador, 1995), and to the demanding nature of women's work (Kellogg, 2005). This intersecting of Wixárika traditions with spatial living arrangements and the selective incorporation of Mexican social arrangements and ‘codes of honour and shame’ (González-López, 2015), has created particular marital structures that are highly unfavourable to women. Today polygyny is supported by the Wixárika political system. It also serves a political purpose, since adult women are defined by their marital status and unmarried women are vulnerable to abuse. Whether polygyny is practised formally or informally, men having multiple partners accentuates an already unequal gender structure. Women are often alone or unsupported at the time of childbirth, and frequently bear the sole responsibility for raising and providing for their families as their husband is occupied with a different wife.

Within this study I did not specifically seek to explore experiences of physical or psychological abuse, but these emerged in subtle ways as women told their birth narratives, in witness accounts that were shared by friends and relatives, and through informants who revealed their own experiences. What became clear is that women are doubly disadvantaged, because traditional authorities sanction and reinforce this violence, which forms part of their subordinate position. The political condonement of intimate partner violence legitimises it in the domestic space. As the case of Maribel demonstrates, there is little appetite within the community to take on a fight against this.

We know that gender relationships in pre-colonial Mexico were largely complementary, meaning that men and women each took on separate household and social tasks that complemented each other (Kellogg, 2005; Stern, 1995). There are plausible explanations for how social/structural changes and events precipitated violence against women. The first of these is that the generalised and widespread use of violence during the colonial period throughout Latin America bred a culture of violence (Segato, 2010; Smith, 2003). Secondly, and linked to this, specific structures of Indigenous masculinity that emerged through interactions between colonisers or mestizo (Mexican) governors and Indigenous men legitimated the use of violence as a form of maintaining order (Morgensen, 2015; Silverblatt, 2009). With reference to previously egalitarian North American communities, Jaimes Guerrero (2003) talks of ‘trickle down patriarchy’, whereby ‘tribal sovereignty’ was legitimised in colonial states through the establishment of patriarchal political orders, effectively implying that Indigenous men took political power away from women, in order to wrest back some form of sovereignty for their communities. Some of these patterns are evident in early twentieth-century Wixárika ethnographies. The introduction of corporal punishment is documented by Lumholtz, 1902, who suggests that its use by governing authorities later became instituted as a form of control over spouses, as the ‘strictness of olden times, becomes obsolete’ (1902: 95). This he refers to as ‘modern improvements’ to aid and abet connubial vagaries, although he makes no specific reference to control over women. In fact, in 1907 Preuss (1996: 114) describes the opposite, the use of a stick to control a *husband's* behaviour. Although references to men beating their wives do appear in these works, on the whole Lumholtz describes the ‘liberal conditions of women’, who are ‘able to decide their own fates’ (1902: 92). While these data must be interpreted in the historical context in which they were written when inequality between men and women was largely considered natural, they nevertheless suggest that the gender dynamic was transformed through the imposition of colonial political and social structures. As links between Wixárika communities and the state were intensified in the post-revolutionary period (1920 onwards), it is possible that the gender dynamic within mestizo culture was further adopted by Wixárika communities. As Kanter (1995: 615) describes, ‘the imposition of a colonial gender order relegated women to a sub-human status firstly as colonial subjects, and secondly as women’. Although Catholicism did not have a major impact on Wixárika *beliefs*, the accompanying political system attached itself to existing religious structures, and brought with it a ‘modern’ system for punishment. The use of violence to correct transgressions at a social and moral level was gradually transformed into intimate partner violence, to ensure that women comply with a specific gender and ‘moral’ order.

In the Wixárika communities that participated in this study, gender inequality appears to lead to preventable forms of maternal and infant mortality, partly through the constricted agency of women in relation to decision-making, but also through the privileged position of men. As was described in the cases of Sarahi and Chavela, the subordinate status of women that positions men as decision-makers can mean that timely decisions about seeking care during childbirth are not made, or are not made in favour of a labouring woman. Some women appeared to be afraid to challenge their husband's authority – a reflection of their subordinate status. Violence is important to this process, as disobedience has become a justification for physical punishment. Research about maternal mortality with Indigenous communities in the south of Mexico has identified similar dynamics (Freyermuth, 2004), but programmes aiming to address maternal and infant health outcomes do not seek to transform gender as a structural inequality; rather, as Wilson (2015) points out, they seek to work within the existing patriarchal system.

Finally, a supportive role of men in childbirth is critical to good outcomes, and the role and decisions men take reflect not only varying forms of gender inequality within relationships, but also the coloniality of care within hospital environments. Highland clinics in Xirawe mostly allow men to be present at the time of birth, but the medical preference for supine delivery denies them active participation. The exclusion of men from hospital births, coupled with the gatekeeping role that *Prospera* plays around pregnancy, birth and childcare, ensures these are defined as women's issues. A systematic review of parenting programmes aimed at fathers identified the fundamental need to understand the gender dimension in parenting programmes, and noted that these should be respectful of cultural values and ‘consonant with structural constraints that shape everyday behaviour’ (Panter-Brick et al., 2014: p22). Several of our informants mentioned the active participation of their husbands, either physically supporting their labouring partners at home and helping to catch the baby/cut the cord, or, where they did not feel able, ensuring their wife seek this support in the clinic. Simultaneously the lack of care in the cases of Chavela and Luisa might indicate a low regard for their wives and consequent disregard for their vulnerability to obstetric complications. Research with Indigenous Mayan and Rarámuri women (Ortega Canto, 2010; Miller, 2009) coincides with the findings of this study that men can play an active and important role in childbirth and that this can lead to positive birth outcomes. Ortega Canto (2010) suggests that such a role can strengthen men's relationship with their children and lead to more equal gender relationships. This is a hypothesis that requires further investigation, but would suggest that decolonising the gender focus of maternal health and actively encouraging men's participation in birth and the care of children could have positive health and gender outcomes.

## 7 Conclusions

Gender structures in Wixárika communities have been built up over time, through the interactions between people and with colonial and post-colonial states. By linking ethnohistory with contemporary ethnographic data, I demonstrate how the coloniality of gender is impacting on maternal and infant health outcomes through institutionalised gender inequality, intimate partner violence and marriage structures that disadvantage women. This is the afterlife of colonialism, the lingering of political domination in the bodies of Indigenous women and infants, resulting in maternal and infant mortality and morbidity.

Decolonial work around gender positions itself as a pathway to greater equality (Lugones, 2010; Wilson, 2015). Jaimes Guerrero (2003) advocates for more historical agency to re-envision pre-patriarchal and pre-colonial society. The ethnohistorical accounts that I have used in this paper require documentary support from archive data, but it is possible that by demonstrating the coloniality of the current gender system, a process of decolonisation can begin. Decolonising gender could bring benefits to health, especially in communities with intersecting burdens of racial and gender inequalities.

In this paper I have emphasised how the Afterlife of Colonialism operates through gender, but it is also a product of racism and ethnic discrimination in and of themselves, and through the coloniality of healthcare. The coloniality of care is evident as Indigenous women who choose an institutional birth have lost control over the way in which they give birth, and their partners are excluded from supporting them in this process. For specific populations whose health is managed by *colonial* care systems, the pursuit of health sovereignty and giving ownership and control over the methods and conditions of healthcare could see increases in skilled attendance, timely access to care and supported environments for birthing. In Mexico some hospitals in Indigenous regions have begun to incorporate traditional medical practices as a subsidiary and marginalised form of care (Gamlin and Berrio, 2020; Dixon et al., 2018), but for Indigenous communities health equity will entail asserting cultural, economic and political control over their health and their healthcare. To achieve this it is necessary to decolonise patriarchal understandings of development, and to disrupt colonial control over Indigenous lives and communities.

## CRediT authorship contribution statement

**Jennie B. Gamlin:** Conceptualization, Data curation, Funding acquisition, Formal analysis, Investigation, Project administration, Methodology, Resources, Software, Supervision, Validation, Writing - review & editing.
